# The healing power of *Camellia japonica* L.: how flower types influence urban residents’ physiological and psychological wellbeing

**DOI:** 10.3389/fpsyg.2025.1489859

**Published:** 2025-02-26

**Authors:** Lijiao Ai, Huan Wang, Yilong Feng, Ting Li, Zezhou Li, Min Zou, Qiaoyong Zhang

**Affiliations:** ^1^Chongqing Landscape and Gardening Research Institute, Chongqing, China; ^2^Chongqing Key Laboratory of Germplasm Innovation and Utilization of Native Plants, Chongqing, China

**Keywords:** *Camellia japonica* L., visual perception, emotions, biological feedback, urban landscape

## Abstract

Color and form are closely related to our daily lives and can directly and rapidly affect people’s emotions, and it is of great significance to study the effects of color and form of garden plants on the body and mind of urban residents. In this study, the shrub *Camellia japonica* L., which has rich germplasm resources, was selected as the research object. It aims to address the following research questions: how flower colors and flower types of *Camellia japonica* L. affect human physiology and psychology? In this study, we recruited 158 participants to participate in a controlled experiment to quantitatively measure and analyze physiological (heart rate, blood pressure, oxygen saturation, electroencephalogram [EEG]) and psychological (anger, panic, nervousness, energy, fatigue, depression, and self-esteem) indices before and after viewing pictures of *Camellia japonica* L. of all colors and flower types, as well as of them in different colony configurations. The results of evaluating physiological indexes and POMS values showed that different types of *Camellia japonica* L. images had different restorative benefits. From the physiological indicators, blood pressure metrics were more sensitive as an evaluation of recovery effects than those of heart rate and oxygen saturation, and stress recovery was more significant after color stimulation than petal category and landscape type. Color, petal type, and landscape configuration type affect relaxation, concentration restoration, and other moods differently reflected by α wave and β wave of EEG. POMS questionnaires showed that viewing different types of picture processes of *Camellia sinensis* significantly reduced nervousness, anger, fatigue, depression, panic, and self-esteem, and the effect was significant in males than in females. Our findings provide a theoretical basis and selection for the garden application of *Camellia japonica* L. in a broader sense, intending to improve their health benefits and maximize the restorative effects of urban environments.

## 1 Introduction

Urban dwellers have faced an increasing number of pressing challenges in recent decades. The rapid expansion of urban populations has become one of the greatest global health challenges of the 21st century (Uriel, 2023). As a result of increasing work pressure and an accelerated pace of life, mental pressure and psychological disorders such as insomnia and anxiety are on the rise (Beyer et al., 2014). There is a higher presence of mental illness in cities, 80% compared with 48% in the countryside, mainly depression, stress, and neurotic disorders, according to a meta-analysis by Reddy and Chandrashekar (1998). According to the World Health Organization, depression has such adverse effects that it has been declared a global epidemic (WHO) (Nieuwenhuijsen et al., 2010).

As part of sustainable development, urban green spaces contribute to the health and wellbeing of individuals. Research has shown that urban residents can benefit physically and psychologically from exposure to the natural environment as a result of those developments (Dong et al., 2021; Wu et al., 2020). Many explanations have been offered for why urban green spaces are beneficial to health. Humans have an innate biological connection to nature, and natural environments restore emotional stability, attention, and stress (Ulrich et al., 1991; Ruoxi, 2016). Following the stress recovery theory (Ulrich et al., 1991), natural elements such as scenes, odors, and sounds can activate the parasympathetic system, which reduces blood pressure, heart rate, skin conductance, and salivary cortisol. It has been suggested that these physiological responses could cause relaxation and reduce autonomic arousal and stress. Later studies have confirmed the possibility of recovering fatigue caused by excessive attention in the natural environment (Lewandowska et al., 2020). Several other pathways have also been investigated that link urban green spaces with human health. Research on the psycho-physiological effects of green spaces has yielded promising results (Yi et al., 2022; Elsadek and Liu, 2021). According to a recent study, viewing the green facade is associated with improved mental and physiological health (Elsadek et al., 2019). Green space access is rapidly declining in urban areas as nature-contact opportunities decrease (Fuller and Gaston, 2009). Recent systematic studies have examined the impact of flower color on emotions and affect. A study by Zhang et al. (2023) with a large sample of 670 participants evaluating eight different flower colors, found that cool flower colors like blue and purple effectively promoted relaxation and reduced stress. In contrast, warm colors like orange, yellow, and red evoked more uplifted and positive emotions (Zhang et al., 2023). These findings were supported by a cross-cultural study conducted in both the UK and USA by Neale et al. (2021). Several experts have increased public awareness of the differences between landscape types (Liu, 2016), landscape elements (White et al., 2010) and spatial characteristics (Peschardt and Stigsdotter, 2013) on health promotion. Some of them even deeply explored the effects of landscape characteristics (Wang et al., 2019) and fine-grained categories of designed urban planting (Wang et al., 2019) on aesthetic preference and perceived restorativeness.

There are numerous uses for plants of the genus Camellia (family Theaceae), from beverages to oils to ornamental plants (Barman et al., 2008; Gui-Ying et al., 2015). The camellia plant is renowned for its large, bright flowers and has been cultivated for thousands of years in China and throughout Asia (Li et al., 2016; Dejun, 1984). In more than a thousand years of camellia cultivation and application, we have made full use of the characteristic resources and cultivated richly colored cultivars with different poses through natural selection (Zhang, 2008). There are nearly more than 250 species and thirty thousand varieties of Camellia in the world, and the characteristics of different varieties are mainly reflected in the flower type and color (Li et al., 2016). Camellia plants are often used in gardens and parks for their beautiful appearance, evergreen, and many varieties. Double flower types of camellias are loved for their fruitful shapes, including a variety of double flower types such as semi-double flower type, peony double flower type, and fully double flower type, but camellias in the wild tend to have simple flowers. Camellia plants not only possess a rich variety of flower types, but the flower color is also an important ornamental trait, of which the red flower type accounts for about 95% or so, and some pink, yellow, and white types. Current research on Camellia focuses on its component research, genetic breeding, and landscape applications et al. (Wang et al., 2020; Lin et al., 2013). It is believed that Camellia’s diverse flower colors and shapes provide a special sense of mental pleasure and reduce potential stress. However, this assessment is entirely subjective. To date, there is insufficient evidence of physical and mental health benefits.

To validate the above belief, a study of the health effects of colors and forms generated by Camellia flowers would be helpful. Furthermore, it will provide useful guidelines for landscape designers on how to enhance the health benefits of urban green spaces by using Camellia species. It aims to address the following two research questions: (1) Do different flower colors and flower types of Camellia plants affect human physiology and psychology? (2) What are the differences in the effects of different flower colors, petal types, and landscape application types? To answer these questions, we conducted a controlled study by exposing 158 participants to different flower colors, petal types, and landscape application types generated by Camellia. The Profile of Mood Stats (POMS) scale and physiological (heart rate, blood pressure, oxygen saturation, electroencephalogram [EEG]) indices were experimented with to measure quantitatively. Using POMS alone may lead to biased results toward the subjective feelings of the subjects (Saddoud et al., 2021). A combination of physiological indices and questionnaires can provide not only subjective information regarding people’s current psychological states but can also provide objective information regarding people’s physiological responses (Li et al., 2020).

Using the combined methods, different flower colors, petal types, and landscape application types of Camellia were investigated on the human body. The results of the research will provide useful information on the use of Camellia species in urban landscapes for better physical and mental health landscapes.

## 2 Materials and methods

### 2.1 Participation

We invited colleagues from our unit and students from cooperative universities to participate in this activity, and gave potted gifts to volunteers who participated in the test. One hundred fifty-eight entirely voluntary participants were recruited for this study, half of the students and the other working populations, aged from 17 to 54 years. Differences in personal liking and professional background may directly affect the experimental objective results. We also collected data on participants’ preferences for pictures. The experimenter’s preference for special colors was collected before and after the experiment and included white, pink, red, and green. For the differentiation and statistics of majors, we include two majors and non-related majors related to garden plants (Table 1). All participants who were undergoing any form of neurological or psychiatric treatment were exempt from participation. To ensure their physical and mental health, participants were asked not to drink alcohol and to keep up a good routine the day before the experiment. Before participating in the study, all subjects provided informed consent.

**Table 1:** Descriptive information of participants who enrolled in the study (*N* = 158).

Characteristic	Variable classification	*N* = 158^*^
Age		17–54 [20, 21]
Sex	Female	116 (73.42%)
	Male	42 (26.58%)
Major (garden plants)	Non-related	75 (47.47%)
	Related	83 (52.53%)
Affiliate institutions	Chongqing Landscape and Gardening Research Institute	50 (31.65%)
	Chongqing Jiaotong University	68 (43.03%)
	Chongqing University of Arts and Sciences	40 (25.32%)
Like_color	Pink	36 (22.78%)
	White	55 (34.81%)
	Red	19 (12.03%)
	Green	48 (30.38%)
Dislike_color	Pink	40 (25.32%)
	White	19 (12.03%)
	Red	61 (38.61%)
	Green	38 (24.05%)

* Median [IQR]; *n* (%).

Upon completion of an informed consent form, all participants were informed regarding the content of the experiment and were told that they had the right to withdraw from it at any time. We followed the National Research Council’s ethical standards and the Helsinki Declaration in all study procedures.

### 2.2 Stimuli

**Figure 1:**
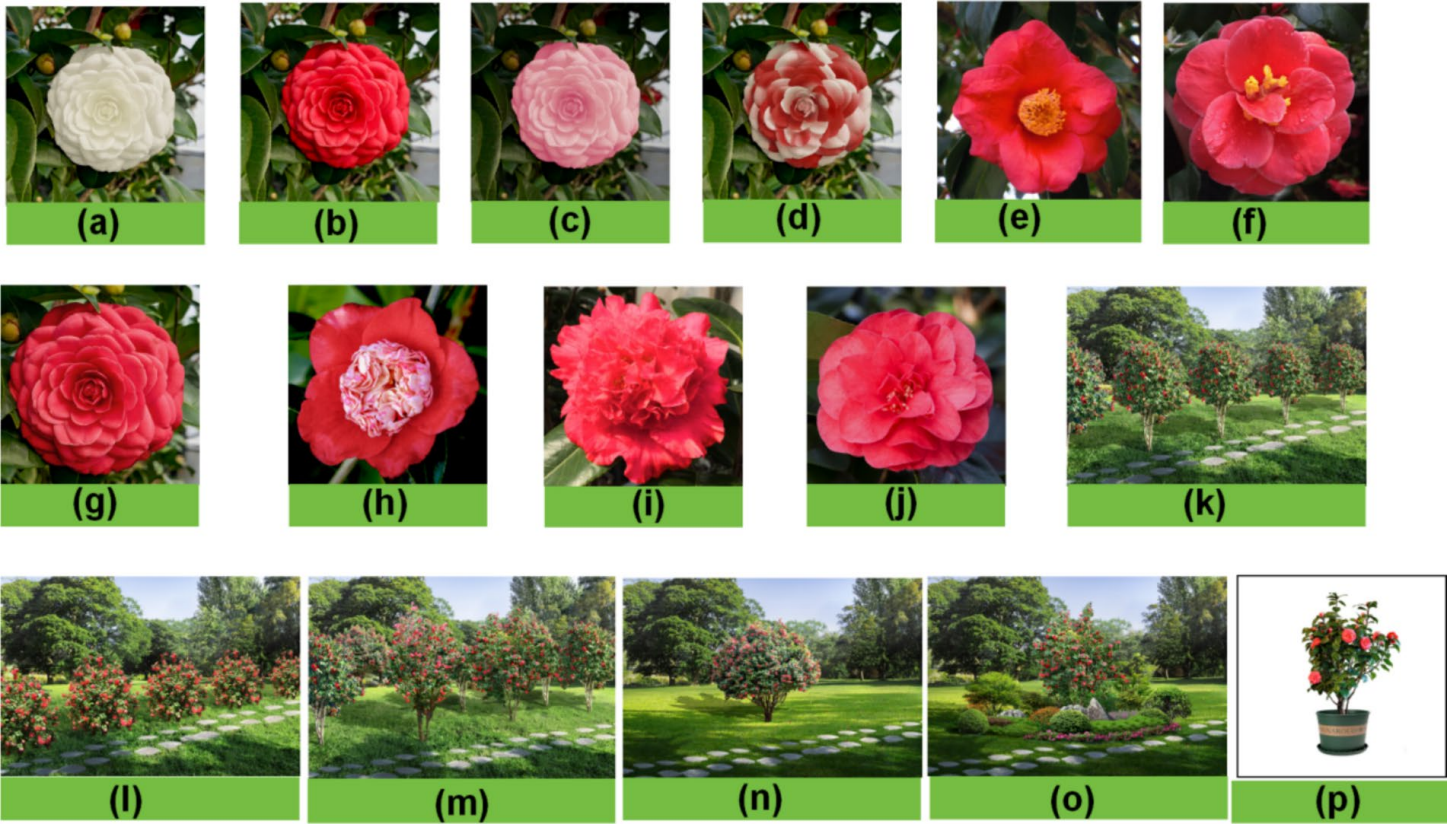
Stimulus images. **(A–D)** group: color group, white **(A)**, red **(B)**, pink **(C)** and variegated **(D)**; **(E–J)** group: flower type group, simple flower **(E)**, semi-double flower type **(F)**, fully double flower type **(G)**, anemone type **(H)**, peony double flower type **(I)** and rose double flower type **(J)**; **(K–P)** group: landscape type group, tree rows **(K)**, shrub rows **(L)**, tree groups **(M)**, tree solitaires **(N)**, clusters **(O)** and potted plants **(P)**.

Camellia species are a rich resource with different flower colors, flower types, and landscape application types. We chose common flower color flower type and landscape type to make stimulus pictures. The pictures in the flower color group (Figures 1A–D) were made into stimulus pictures with different flower colors but the same flower type and background using Photoshop software. Pictures in the flower type group (Figures 1E–J) were created as stimulus pictures with different flower colors but the same flower color and background using Photoshop software. Pictures in the landscape type group (Figures 1K–P) used Photoshop software to make the pictures into stimulus pictures with different flower types landscape types and the same background. The visual characteristics of other elements in each group environment were identical.

### 2.3 Experimental procedure

**Figure 2:**
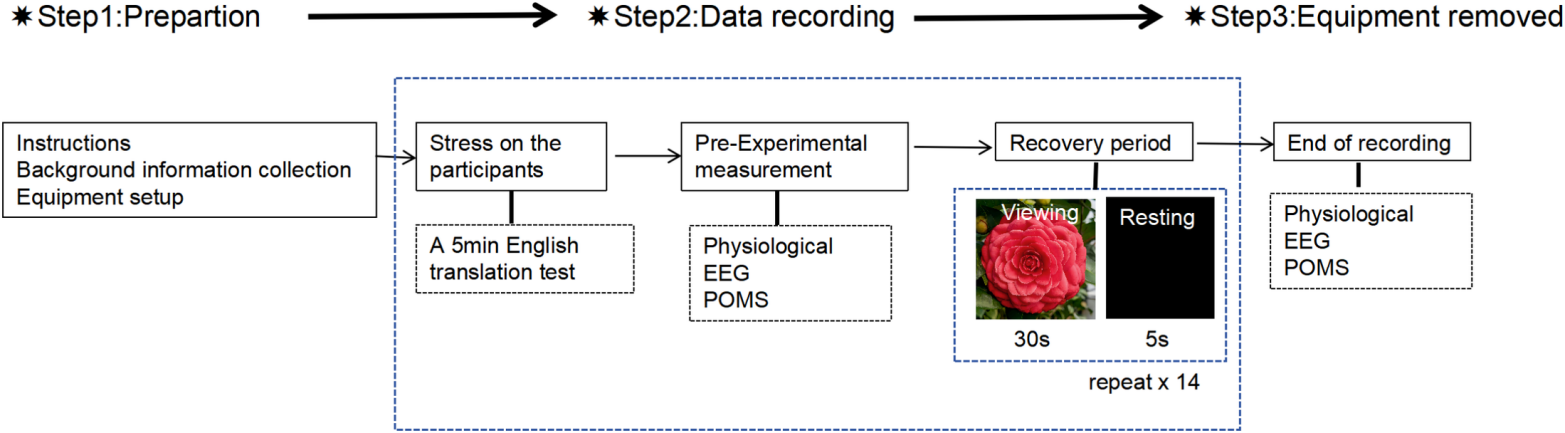
Experimental procedure.

During the experiment, each subject participated individually and each experiment lasted about half an hour. The procedure is shown in Figure 2. The staff distributed number cards to the participants, and groups of three entered the test room to prepare. Staff put on EEG, blood pressure, and oximeter to the subjects. Subjects were given a stress test in the classroom and were given 5 min to complete the English translation test questions. The staff timed the reminder, and after the 5-min stress test, their physiological and electroencephalographic values after the stress test were measured and recorded. At the same time, the subjects used their mobile phones to scan and fill in the Pre-Experimental Mood State Scale (short form POMS). Cycle with images staying for 30 s and a black screen for 5 s for a class of images, the pictures shown in sequence are Flower-White, Flower-Red, Flower-Pink, Flower Color-Variegated, Flower type-single, Flower type-semi-double, Flower type-fully double, Flower type-anemone, Flower type-peony double, Flower type-rose-double, Landscape-tree rows, Landscape-shrub rows, Landscape-tree groups, Landscape-tree solitaires, Landscape-clusters, Landscape-potted plants. The staff recorded the physiological and electroencephalographic measurements of the subjects after looking at each type of picture within 5 s of the black screen. After subjects finished viewing the 16 camellia pictures, they used their mobile phones to scan and fill in the Post-Experimental Mood State Scale (short form POMS).

### 2.4 Measurement items

#### 2.4.1 Physiological stress

In this study, the biofeedback measurement method was adopted, and blood pressure (diastolic blood pressure, SBP for short; systolic blood pressure, DBP for short), heart rate (HR for short), and blood oxygen saturation (SpO_2_ for short) were selected as indicators of physiological changes in participations. Blood pressure and heart rate were measured high-precision electronic sphygmomanometer (Yuwell, 670A, China); blood oxygen saturation was measured by a finger-clip finger oximeter (Yuwell, YX102, China). Using a Neurosky mindwave EEG headset (Beijing Oriental Creation Technology Co., Ltd., China), we measured EEG signals transmitted from the forehead (Deng et al., 2019). It was light and compact and did not cause obvious discomfort to users. The headset had four essential components: (1) a sensor arm containing the EEG electrode, (2) a Bluetooth module, (3) a headband, and (4) an ear clip. The α wave and β wave are generally thought to display the closest correlation with human emotions. The alpha waves (8–12 Hz) are the gateway to meditation, relaxation, open-eyes dreaming, learning, the feeling of being present. They originate in the occipital area and advance to the frontal sides of the brain that process emotion and behavior. High alpha intensities (approximately 10–12 Hz) are correlated with relaxation. The beta waves (12–30 Hz) are responsible for states of attention, alertness, cognition, decision-making and problem-solving (Oana et al., 2019; Coben et al., 2010; Robbins and Stonehill, 2014). We used the accompanying analysis software Mindwave Mobile and Visualizer for EEG data processing. This software sorts brain wave signals from weak to strong on a scale of 1–100 during states of relaxation and attention (Adem et al., 2017).

#### 2.4.2 Psychological tests

Psychological tests were used, and the Profile of Mood States (POMS) was chosen to measure and evaluate the psychological indicators of participants, with higher scores representing higher anxiety and vice versa. The POMS questionnaire consists of 40 questions and is divided into seven dimensions: tension, anger, fatigue, depression, vigor, panic, and self-esteem, with each question scored from 1 to 10, and the higher the score for each dimension, the higher the degree of emotion. The questionnaire was presented in Supplementary Table S1.

### 2.5 Data analysis

SPSS 25.0 (IMB SPSS Statistics) was used to process the experimental data and calculate the differences in physiological, electroencephalographic, and psychological data between the subjects after the viewing activity and the stress control group. All data were tested for normal distribution using the Shapiro–Wilk test (Royston, 1992). Pre and post paired t-test (Paired sample Test) was used to compare the changes in the mean values of physiological, EEG, and psychological indices and the paired differences were computed by subtracting the differences between the pre and post experiment. The Wilcoxon signed rank test was used to analyze the POMS data. The threshold for statistical significance was set at *p* < 0.05.

## 3 Results

### 3.1 The effects of color, type, and landscape type on the physiological index

Did the color, type, and landscape type influence participants’ physiological stress? The paired difference (pre-stimulus minus post-stimulus) and *p*-value significance of the heart rate, systolic blood pressure, diastolic blood pressure, and SpO_2_ data are shown in Table 2. In general, among the different physiological index tests, blood pressure (telescopic and diastolic) was more sensitive than heart rate and blood oxygen saturation. In terms of Systolic Pressure, all color, petal, and landscape types have a significant recovery effect. Of the results for diastolic blood pressure, only landscape-shrub rows did not have a significant recovery effect (*p* > 0.05), while all other types had a significant recovery effect. After the different pictures stimulus experiment, the mean heart rate of four colors exhibited a significant (2.85 ± 1.97, 2.67 ± 2.07, 1.81 ± 1.78, and 2.35 ± 0.80, *p* < 0.01); while neither the flower types and Landscape types showed a significant effect on heart rate. Similarly, the result further tested the effects related to blood oxygen saturation (SpO_2_). The results showed that only the color-related stimuli (red and pink) had a significant effect on blood oxygen saturation and the pairwise differences for red and pink are 0.83 ± 0.68 and 1.02 ± 1.08, respectively (*p* < 0.01). Flower color showed significance in both heart rate and blood pressure, while flower pattern and landscape type were only significant in blood pressure (Table 2), Overall, between the different stimulus picture types, color was more sensitive than the petal type and landscape type.

**Table 2:** Heart rate, systolic pressure, diastolic pressure, and SpO_2_ after each type of picture stimulation.

Category	Heart rate	Systolic pressure	Diastolic pressure	SpO_2_
1. Flower-White	2.85 ± 1.97**	3.36 ± −0.43**	2.79 ± 0.16**	0.26 ± 0.11
2. Flower-Red	2.67 ± 2.07**	6.62 ± 2.71**	4.49 ± 0.22**	0.83 ± 0.68**
3. Flower-Pink	1.81 ± 1.78*	6.62 ± 0.73**	6.01 ± 0.51**	1.02 ± 1.08**
4. Flower -Variegated	2.35 ± 0.80**	7.59 ± 2.44**	6.55 ± 0.63**	−3.85 ± 63.77
5. Flower type-single	1.01 ± 1.51	8.19 ± 1.02**	6.95 ± 0.17**	0.51 ± 0.19
6. Flower type-semi-double	0.95 ± 1.10	8.88 ± 1.10**	8.45 ± 0.20**	0.86 ± 4.16
7. Flower type-fully double	0.23 ± 1.31	8.56 ± 0.58**	6.09 ± 29.24*	0.39 ± 0.02
8. Flower type-anemone	−0.62 ± 1.32	9.60 ± 0.86**	8.88 ± 0.20**	0.32 ± 0.38
9. Flower type-peony double	0.26 ± 1.52	10.06 ± 1.45**	6.65 ± 29.58	1.05 ± 4.36
10. Flower type-rose-double	0.63 ± 1.61	10.22 ± 0.46**	10.30 ± 0.16**	0.49 ± 0.12
11. Landscape-tree rows	−0.26 ± 1.61	11.51 ± 2.31**	10.85 ± 2.51**	0.62 ± 0.2**
12. Landscape-shrub rows	−0.49 ± 1.46	12.99 ± 2.4**	6.57 ± 36.95	0.96 ± 5.00
13. Landscape-tree groups	−0.14 ± 1.65	10.91 ± 1.35**	8.13 ± 22.31**	0.31 ± 0.37
14. Landscape-tree solitaires	−0.30 ± 2.11	11.18 ± 0.26**	7.78 ± 30.51*	0.70 ± 4.11
15. Landscape-clusters	−0.68 ± 1.61	11.47 ± 0.52**	11.32 ± 0.96**	0.20 ± 0.37
16. Landscape-potted plants	−0.94 ± 1.71	12.12 ± 1.16**	10.86 ± 1.52**	0.22 ± 0.24
Color	2.17 ± 2.25*	3.9 ± −0.237**	2.6 ± 0.582**	−0.01 ± 0.051
Flower type	0.81 ± 2.288	8.87 ± 1.455**	7.32 ± −0.436**	0.42 ± −0.252
Landscape	−0.91 ± 2.191	12.8 ± −4.142**	10.84 ± −1.994**	0.23 ± 0.053

*p < 0.05; **p < 0.01.

### 3.2 The effects of color, type, and landscape type on focus and relaxation, as reflected by the brain waves

**Figure 3:**
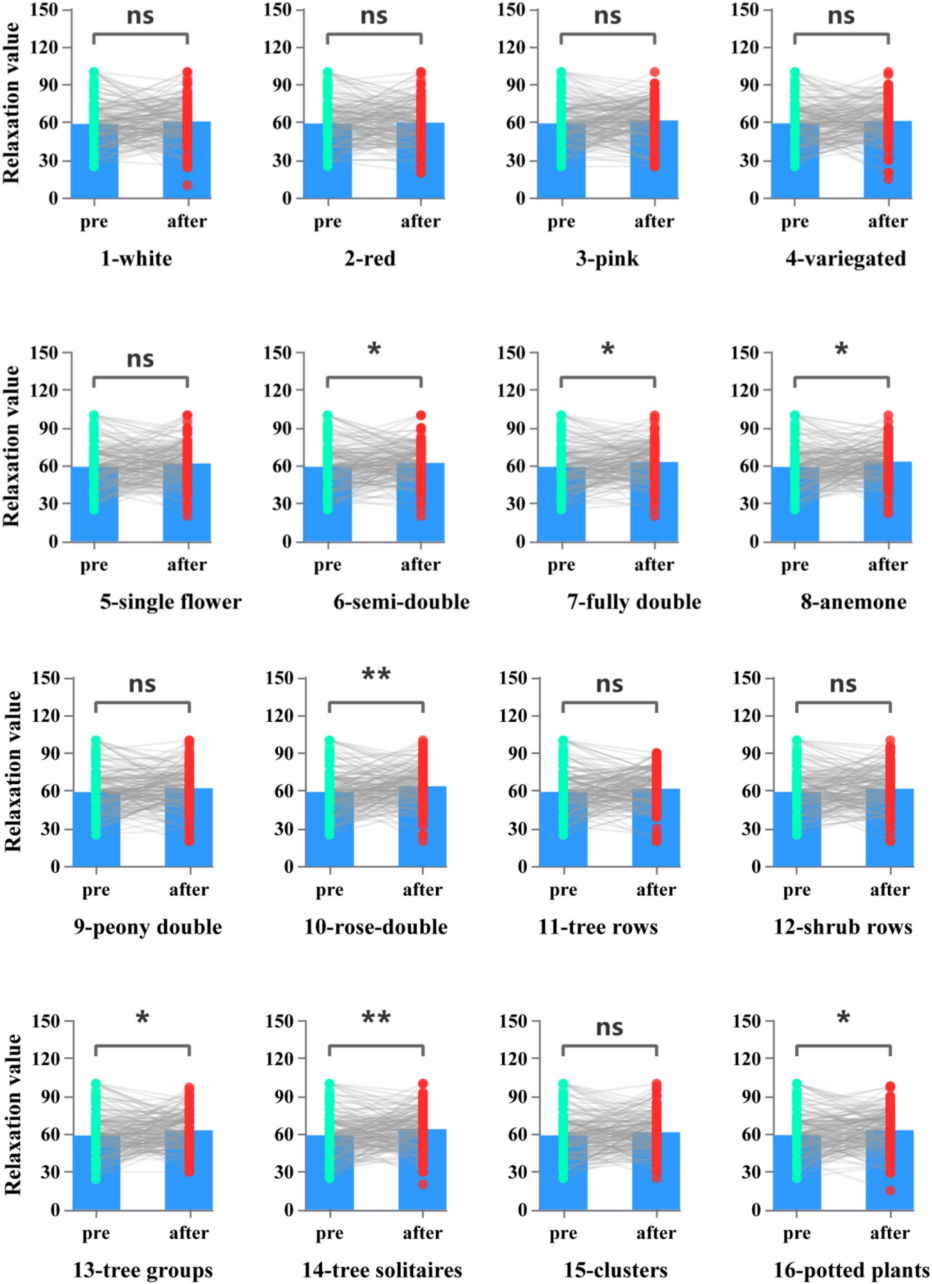
Relaxation values after each type of picture stimulation in response to brain wave testing.

We compared the EEG alpha frequency and high beta wave while the participants were watching the slide show, in which alpha frequency is associated with a state of alert relaxation and beta frequency with increased concentration. The higher the EEG-Alpha, the less arousal and stress were measured. Paired t-test showed that pictures 6 (Flower type-semi-double), 7 (Flower type-fully double), 8 (Flower type-anemone), 10 (Flower type-rose-double), 13 (Landscape-tree groups), 14 (Landscape-tree solitaires), and 16 (Landscape-potted plants) relaxation value (alpha frequency reflecting) was significantly higher than the other group (Figure 3, *p* < 0.05). Color stimulation did not show a significant effect on the alpha-wave. The effect of flower type (Semi-double flower type, fully-double flower type, anemone flower type, rose-double flower type) stimulation on relaxation was even more pronounced. The effect of landscape-type visual stimulation on relaxation also varied by planting method, in which tree groups, tree solitaires, and potted plants showed significant differences.

**Figure 4:**
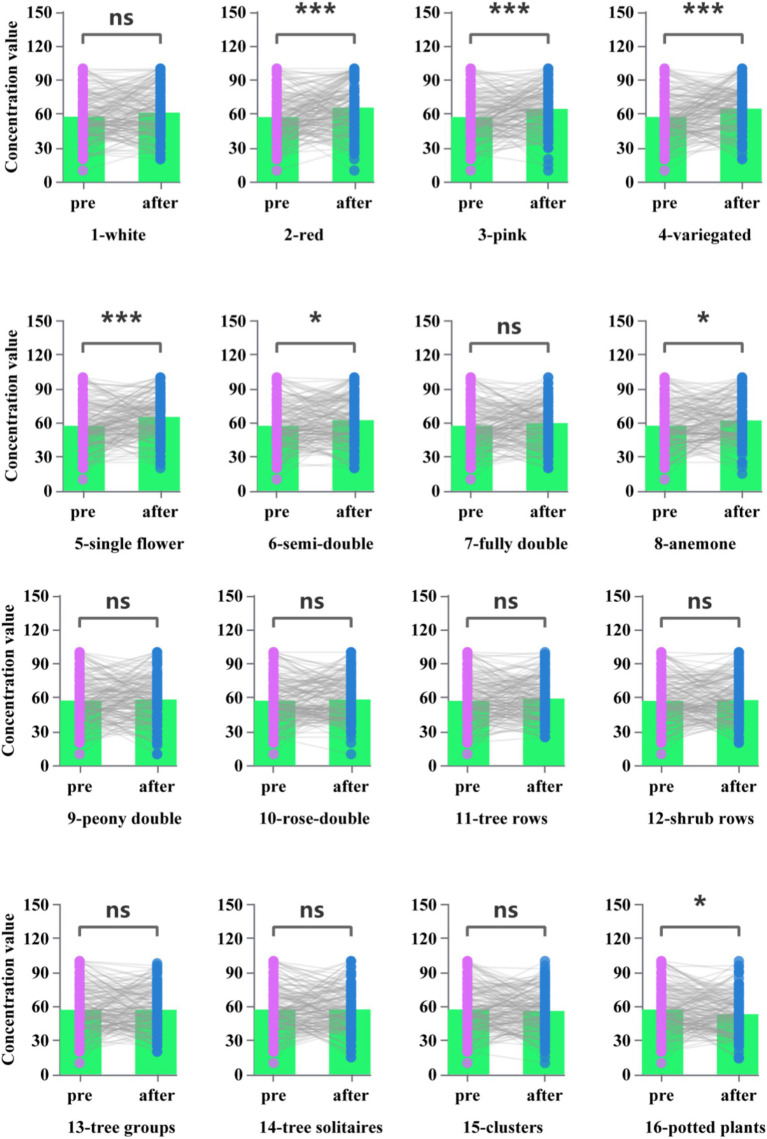
Concentration values after each type of picture stimulation in response to brain wave testing.

The higher the EEG-beta, the less concentration value was measured. Paired t-test showed that the pictures 2 (Flower-Red), 3 (Flower-Pink), 4 (Flower-Variegated), 5 (Flower type-single), 6 (Flower type-semi-double), 8 (Flower type-anemone), and 16 (Landscape-potted plants) concentration value (beta frequency reflecting) was significantly higher than the other group (Figure 4, *p* < 0.05). Overall, flower color type had a higher effect on concentration value than petal type and landscape combination type.

### 3.3 Psychological effects

#### 3.3.1 The effects of color, petal, and landscape type on mood

**Figure 5:**
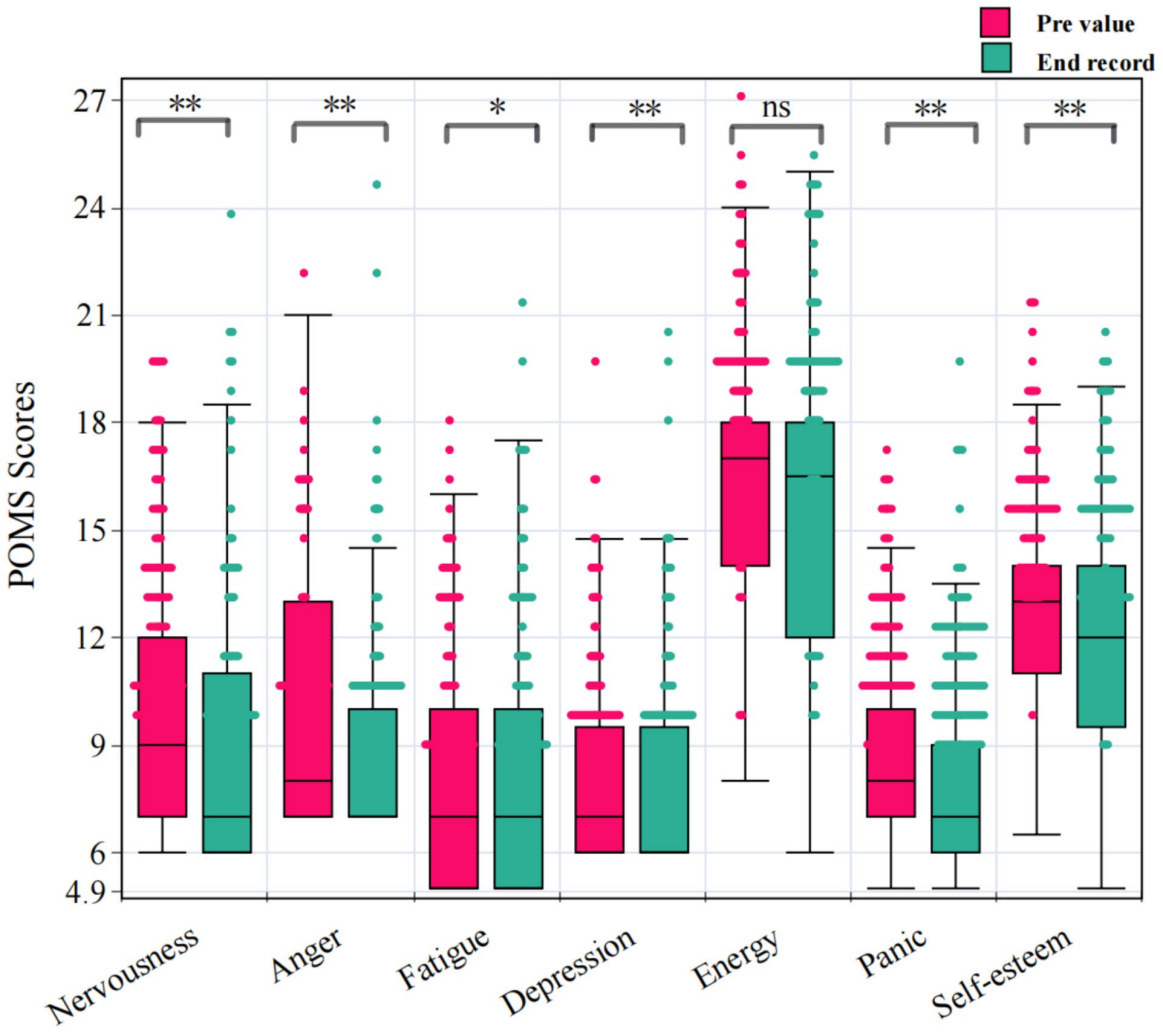
POMS scores of different psychological conditions.

Profile of mood states (POMS) questionnaires were used to assess the psychological responses of participants to the stimuli procedure. Paired samples t-test analysis of the Profile of mood states (POMS) questionnaires before and after the stimulation process showed that viewing different types of picture processes of *Camellia sinensis* significantly reduced nervousness, anger, fatigue, depression, panic, and self-esteem, as shown in Figure 5 (*p* < 0.05), with paired differences of 1.103 ± 0.091, 0.747 ± 0.562, 0.505 ± 0.123, 0.758 ± 0.618, 1.206 ± 0.164, and 0.66 ± 0.356, respectively. However, there was no significant difference in energy recovery (*p* > 0.05). In terms of the magnitude of the paired differences, the effect of the stimulation process was most pronounced on the effects of nervousness and Panic.

#### 3.3.2 The difference in the influence of gender and professional major on experimental mood results

As shown in Table 3, there were significant differences after the stimulus recovery process of images. The mean restorative value of psychological indicators of women showed less obvious beneficial changes. Males showed significant validity in nervousness, anger, energy, and self-esteem (*p* < 0.05), while females showed significant validity only in energy and self-esteem (*p* < 0.05). From the analysis of the sub-specialization categories, Differences between specialties fields were not significant, and similarly, the indicators with significant validity were both energy and self-esteem.

**Table 3:** Mean restorative value of psychophysiological responses of different genders and major for stimulation process.

Psychological indexes	Significance of the pairing difference value		Significance of the pairing difference value	
	Gender-male	Gender-female	Major-non-related garden plants	Major-related garden plants
Nervousness	1.44 ± 0.07 (0.018**)	0.27 ± 0.47 (0.356)	0.63 ± 0.72 (0.093)	0.47 ± 0.22 (0.194)
Anger	1.47 ± 1.21 (0.047*)	0.08 ± 0.52 (0.832)	0.40 ± 0.23 (0.327)	0.75 ± 0.29 (0.214)
Fatigue	0.79 ± 0.74 (0.279)	−0.03 ± 0.35 (0.930)	−0.07 ± 0.59 (0.863)	0.54 ± 0.25 (0.340)
Depression	0.61 ± 0.36 (0.319)	0.23 ± 0.12 (0.464)	−0.06 ± 0.43 (0.879)	0.88 ± 0.30 (0.064)
Energy	1.39 ± 0.62 (0.011*)	1.05 ± 0.28 (0.016*)	1.01 ± 0.64 (0.008**)	1.54 ± 0.32 (0.026*)
Panic	1.00 ± 0.48 (0.074)	0.08 ± 0.31 (0.779)	0.47 ± 0.06 (0.117)	0.30 ± 0.03 (0.512)
Self-esteem	0.88 ± 0.55 (0.027*)	1.06 ± 0.25 (0.000**)	0.95 ± 0.40 (0.000**)	1.07 ± 0.24 (0.002**)

*p < 0.05; **p < 0.01.

## 4 Discussion

The study objective was to identify the relationship between physiological, psychological, and restorative benefits when viewing Camellia images with different colors, petal types, and landscape configuration types. As a result, we examined the influence of spatial characteristics and environmental components on environmental restorative effects. In accordance with previous studies (Stigsdotter et al., 2017; Gidlow et al., 2016), the current results confirmed that urban green space would have positive restorative effects on human physiological and psychological wellbeing. Comparing restorative differences between different image characteristics, POMS scores, and physiological indices both showed differences. Despite this, the results of evaluating POMS values and physiological indexes showed that different types of Camellia images had different restorative benefits.

### 4.1 Color, petal type, and landscape configuration type affect physiological stress differently

Exposure to the natural view of the city has been found to have a number of health benefits in previous studies. A study conducted by Mao et al. (2012) found that forests can prevent cardiovascular disease and have therapeutic effects on hypertension in humans. People’s physiological (heart rate, blood pressure, EEG, etc.) and psychological (anxiety, depression, attention, etc.) indicators show significant changes when they view or experience the natural environment (Vujcic et al., 2019). All types of picture stimuli in this study had a significant effect on blood pressure, and overall, blood pressure metrics were more sensitive as an evaluation of recovery effects than those of heart rate and oxygen saturation. Stress or relaxation can affect the sympathetic and parasympathetic nervous systems in the human body, resulting in changes in blood pressure and heart rate (Deng and Deng, 2018). Blood pressure and pulse rate are raised by the stress emotion, whereas they are decreased by the relaxed emotion, as described in the literature (Zanstra and Johnston, 2011; Ahmad et al., 2018). When the organism is in a state of stressful activity, sympathetic activity plays a major role. Accordingly, the different Camellia images were supposed to be able to regulate the sympathetic and parasympathetic nervous systems, as well as improve the participants’ emotional states. From the physiological indicators, stress recovery was more significant after color stimulation than petal category and landscape type. Furthermore, when people appreciate plants, they immediately notice their color, which has a profound effect on their reaction (Zhang et al., 2012). It has been found that flowers of different flowering periods or different colors influence psychological responses in different ways (Zhao et al., 2019; Kim and Fujii, 1995).

### 4.2 Color, petal type, and landscape configuration type affect relaxation, concentration restoration, and other moods differently

Our results show that the mean relaxation value and concentration values of the experimental groups differed differently for different categories of picture stimuli, which responded from brainwave test results. The presence of alpha waves is related to reduced mental stress, increased relaxation, and improved memory (Alarcão and Fonseca, 2017; Ismail, 2020). There is also evidence that exposure to the natural environment leads to increased alpha wave strength, which plays an important role in physiological relaxation and recovery (Kim et al., 2013; Ursuiu et al., 2018). In this research, flower petal types responded more significantly to (relaxation value) a-waves than to color and landscape categories. Our study is similar to the findings of several previous studies on the benefits of plant shapes for health, healing, and mood regulation, such as the stem and leaf shapes of bamboo (Wang et al., 2021). One of the suggestions for this result is to add flowering plants with diverse petal types to places where relaxation is needed, such as studios, living rooms, hospitals, etc. In terms of concentration values, flower color sensitivity is higher than the petal type and landscape type. This is similar to the case for physiological indicator responses. In a study by Zhang et al. (2023) in which 670 participants evaluated eight different flower colors, warm colors, such as orange, yellow, and red, evoked more positive feelings, while cool colors like blue and purple were found to promote relaxation and reduce stress in participants (Zhang et al., 2023). The choice of flower colors in the experiment was dominated by warm colors, which is similar to their findings. It was found that attention and relaxation scores in the experimental condition (Flower type-semi-double and anemone) were both significantly higher than those in the control condition. There is a tendency to infer from this that states of relaxation and concentration can occur simultaneously to some degree. The findings are in line with research on emotional changes caused by environmental contact, such as virtual visual stimulation experiments, walking in bamboo forests and cities, and horticultural activities (Ahmad et al., 2018; Guo et al., 2019; Hassan, 2018). In this respect, it might be suggested that the use of warm-colored flowering plants at the workplace can improve the efficiency and concentration levels of workers. According to the POMS questionnaire results, most of the average scores on each scale improved both in control and experimental conditions, with some changes being significant. Different camellia pictures stimulated a better emotional response. Previous studies have found that flowers of different flowering periods or different colors influence psychological responses in different ways (Zhao et al., 2019; Kim and Fujii, 1995). Because filling out the self-report scale was done after looking at all types of stimulus pictures, it was more significant in terms of the psychological stress recovery effect than the physiological response after looking at each picture. This may be related to the total effective time of looking at the stimulus pictures.

### 4.3 Gender and major background influence peoples’ preferences and recovery effects

The stress recovery experiment in this study involves two main subjects, one is the stimulus picture and the other is the variability of the subject population. There are relevant studies showing that demographic variables, such as age, ethnicity, nature-relatedness, and educational background, are strongly related to general preferences (Zena, 2011; Ghamari and Amor, 2016). A notable characteristic of the strongest responses was that they were associated with colors, petals and landscape types that participants actually liked. In this study, males had a more significant recovery effect than females. Moreover, body hormones affected females’ antistress ability in a significantly different way from males (Guo et al., 2022). Men and women experience different physiological responses to acute stress (Brigitte and Kudielka, 2005), which impacts their cognition and emotion (Jentsch et al., 2022). Evidence suggests that stress increases men’s skills in downregulating negative emotions, while women do not experience this effect (Langer et al., 2023; Langer et al., 2020). In line with expectations, raising cortisol levels pharmacologically resulted in reduced emotional responses (Jentsch et al., 2019) and heightened dorsomedial prefrontal cortex activity in men, but not in women, when they viewed emotional images (Ma et al., 2017). These data suggest that men profit more from the beneficial effects of stress on ER than women (Hamza et al., 2024). The result will be more willingness and ability on the part of most males to actively engage in work. An explanation can be put forward here that visiting a series of camellia picture experiments is a more pleasurable thing for men, and therefore stress recovery is better for men as compared to women. In terms of professional background differences, there was no significant difference in whether it was related with garden plants and this result was unexpected to us. In terms of Energy and Self-esteem restoration, there were significant restoration effects with or without a background in landscaping plants.

### 4.4 Limitations and future research

A number of limitations were identified in this study. First, only typical Camellia strains were used as experimental material, and the experiments focused solely on their color, petal type, and landscape configuration, Generalizability may thus be limited. It is recommended that further research be conducted using other experimental plants and a wider range of colors in order to reach more general conclusions. Additionally, the stimulation time of images in this study was relatively short. It may be possible in the future to use real plant materials or 3D displays to extend the simulation time. Third, as mentioned in the methods section, POMS was not tested after each picture due to the long time of the psychological questionnaire. Future studies should reasonably consider the questionnaire time and the recovery time. All of the participants in this study consisted mainly of students and young researchers, whose professional and age ranges are rather restricted, and a more diverse testing population could be considered in the future.

## 5 Conclusion

Color, petal type and landscape combination of plants can potentially increase the health benefits delivered by the green spaces. This study investigated the effects of color, petal type and landscape combination of *Camellia japonica* L. on participants’ preferences as well as their physiological and psychological responses. Our findings provide preliminary evidence to include *Camellia japonica* L., and flowering woody plants species in a broader sense, in urban green spaces. The combination of physiological and psychological indicators has led to better understanding of the health effects of the plant. The method can be used in future studies of other flowering woody plants. Future research is also needed to explore a more rigorous method to avoid single sensory experiments and to consider the complex factors that influence the emotional changes of people’s perception of vision. By considering the health implications of *Camellia japonica* L. and other flowering woody plants species in a broader sense, urban administrative staff and planners can make better decisions to enhance the ecological and human health of the design.

## Data Availability

The raw data supporting the conclusions of this article will be made available by the authors without undue reservation.
