# Supplementary material for: The healing power of Camellia japonica L.: how flower types influence urban residents’ physiological and psychological wellbeing
Source: Front Psychol. 2025 Feb 26;16:1489859. doi: 10.3389/fpsyg.2025.1489859 (PMC11897270; doi:10.3389/fpsyg.2025.1489859)
Supplement: Supplementary file 1 [file Table_1.docx]

**Supplementary table 1. The Profile of Mood Stats (POMS)**

（Please choose an answer that is most consistent with your situation according to your actual feelings）

| 1. Number: |  |  |  |  |
| --- | --- | --- | --- | --- |
| 2. Name: |  |  |  |  |
| 3. Your gender: |  |  |  |  |
| 4. Age: |  |  |  |  |
| 5. Professional |  |  |  |  |
| 6. Favorite color |  |  |  |  |
| White | Pink | Red | Green |  |
| 7.Colors you don't like |  |  |  |  |
| White | Pink | Red | Green |  |
| 8.Nervous |  |  |  |  |
| Hardly | A little | Moderate | More | Very much |
| 9. Angry |  |  |  |  |
| Hardly | A little | Moderate | More | Very much |
| 10. Lethargic |  |  |  |  |
| Hardly | A little | Moderate | More | Very much |
| 11. Unhappy |  |  |  |  |
| Hardly | A little | Moderate | More | Very much |
| 12. Relaxed and happy |  |  |  |  |
| Hardly | A little | Moderate | More | Very much |
| 13. Flustered |  |  |  |  |
| Hardly | A little | Moderate | More | Very much |
| 14. Embarrassed |  |  |  |  |
| Hardly | A little | Moderate | More | Very much |
| 15. Upset |  |  |  |  |
| Hardly | A little | Moderate | More | Very much |
| 16. Angry |  |  |  |  |
| Hardly | A little | Moderate | More | Very much |
| 17. Tired |  |  |  |  |
| Hardly | A little | Moderate | More | Very much |
| 18. Sad |  |  |  |  |
| Hardly | A little | Moderate | More | Very much |
| 19. Energetic |  |  |  |  |
| Hardly | A little | Moderate | More | Very much |
| 20. Can't concentrate |  |  |  |  |
| Hardly | A little | Moderate | More | Very much |
| 21. Self-confident |  |  |  |  |
| Hardly | A little | Moderate | More | Very much |
| 22. The heart is uneasy |  |  |  |  |
| Hardly | A little | Moderate | More | Very much |
| 23. Annoyed |  |  |  |  |
| Hardly | A little | Moderate | More | Very much |
| 24. Exhausted |  |  |  |  |
| Hardly | A little | Moderate | More | Very much |
| 25. Frustrated |  |  |  |  |
| Hardly | A little | Moderate | More | Very much |
| 26. Be proactive |  |  |  |  |
| Hardly | A little | Moderate | More | Very much |
| 27. Panicked |  |  |  |  |
| Hardly | A little | Moderate | More | Very much |
| 28. Restless |  |  |  |  |
| Hardly | A little | Moderate | More | Very much |
| 29. Annoying |  |  |  |  |
| Hardly | A little | Moderate | More | Very much |
| 30. Burnout |  |  |  |  |
| Hardly | A little | Moderate | More | Very much |
| 31. Melancholy |  |  |  |  |
| Hardly | A little | Moderate | More | Very much |
| 32. Interested |  |  |  |  |
| Hardly | A little | Moderate | More | Very much |
| 33. Forgetful |  |  |  |  |
| Hardly | A little | Moderate | More | Very much |
| 34. Have a sense of ability |  |  |  |  |
| Hardly | A little | Moderate | More | Very much |
| 35. Easily excited |  |  |  |  |
| Hardly | A little | Moderate | More | Very much |
| 36. Angry |  |  |  |  |
| Hardly | A little | Moderate | More | Very much |
| 37. Exhausted |  |  |  |  |
| Hardly | A little | Moderate | More | Very much |
| 38. Worthless |  |  |  |  |
| Hardly | A little | Moderate | More | Very much |
| 39. Energetic |  |  |  |  |
| Hardly | A little | Moderate | More | Very much |
| 40. There is a sense of uncertainty |  |  |  |  |
| Hardly | A little | Moderate | More | Very much |
| 41. Satisfied |  |  |  |  |
| Hardly | A little | Moderate | More | Very much |
| 42. Worried |  |  |  |  |
| Hardly | A little | Moderate | More | Very much |
| 43. Fury |  |  |  |  |
| Hardly | A little | Moderate | More | Very much |
| 44. Complaining |  |  |  |  |
| Hardly | A little | Moderate | More | Very much |
| 45. Lonely and helpless |  |  |  |  |
| Hardly | A little | Moderate | More | Very much |
| 46. Full of energy |  |  |  |  |
| Hardly | A little | Moderate | More | Very much |
| 47. Proud |  |  |  |  |
| Hardly | A little | Moderate | More | Very much |
